# Excitation Wavelength and Intensity-Dependent Multiexciton Dynamics in CsPbBr_3_ Nanocrystals

**DOI:** 10.3390/nano11020463

**Published:** 2021-02-11

**Authors:** Chaochao Qin, Zhinan Jiang, Zhongpo Zhou, Yufang Liu, Yuhai Jiang

**Affiliations:** 1 Henan Key Laboratory of Infrared Materials & Spectrum Measures and Applications, School of Physics, Henan Normal University, Xinxiang 453007, China; qinch@hotmail.com (C.Q.); znjiang0418@163.com (Z.J.); yf-liu@henannu.edu.cn (Y.L.); 2 Shanghai Advanced Research Institute, Chinese Academy of Sciences, Shanghai 201210, China

**Keywords:** Auger recombination, hot-exciton cooling, transient absorption spectroscopy, temperature-dependent photoluminescence spectroscopy

## Abstract

CsPbBr${}_{3}$ has attracted great attention due to unique optical properties. The understanding of the multiexciton process is crucial for improving the performance of the photoelectric devices based on CsPbBr${}_{3}$ nanocrystals. In this paper, the ultrafast dynamics of CsPbBr${}_{3}$ nanocrystals is investigated by using femtosecond transient absorption spectroscopy. It is found that Auger recombination lifetime increases with the decrease of the excitation intensity, while the trend is opposite for the hot-exciton cooling time. The time of the hot-carriers cooling to the band edge is increased when the excitation energy is increased from 2.82 eV (440 nm) to 3.82 eV (325 nm). The lifetime of the Auger recombination reaches the value of 126 ps with the excitation wavelength of 440 nm. The recombination lifetime of the single exciton is about 7 ns in CsPbBr${}_{3}$ nanocrystals determined by nanosecond time-resolved photoluminescence spectroscopy. The exciton binding energy is 44 meV for CsPbBr${}_{3}$ nanocrystals measured by the temperature-dependent steady-state photoluminescence spectroscopy. These findings provide a favorable insight into applications such as solar cells and light-emitting devices based on CsPbBr${}_{3}$ nanocrystals.

## 1. Introduction

Nanocrystal quantum dots (QDs) are intensively studied for the next-generation optoelectronic materials [1]. Cesium lead halides QDs in the form of CsPbX${}_{3}$ (X = Cl, Br, or I) show attractive properties such as tunable bandgaps, ultrahigh photoluminescence (PL) quantum yields, wide tunable emission range, high defect tolerance, and low-threshold optical gain [2,3,4]. With these exciting properties, CsPbX${}_{3}$ semiconductors have emerged as promising materials in applications such as light-emitting diodes (LEDs), lasers, photodetectors, and solar cells [5,6,7].

In traditional semiconductors, photogenerated energetic carriers, i.e., hot-carriers or hot-excitons, relax rapidly to band edges by the emission of phonons [8,9]. The multiexciton generation is a process in which two or even more electron–hole pairs are created in nanostructured semiconductors by absorbing a single high-energy photon [10]. It is difficult to harvest the excess energy above the band edges of the hot carriers due to the carrier relaxation that usually occurs on a sub-picosecond timescale [8]. Quantum confined semiconductor nanocrystals (NCs), such as CsPbX${}_{3}$ NCs, are predicted to have long-lived hot-carriers enabled by a phonon bottleneck where the large inter-level spacings in NCs result in inefficient phonon emissions. The long lifetimes of hot-excitons provide an opportunity for their extraction. However, there is another relaxation process—non-radiative Auger recombination (AR)—in CsPbBr${}_{3}$ NCs. In the AR process, electrons and holes recombine directly, transferring their energy to a third particle which would be re-excited to a higher energy state [11,12]. The exciton energy is lost in the AR, which hampers the PL efficiency and is disadvantageous for applications that rely on light emission [13]. Therefore, it is critical to understand the mechanism of the multiexciton process in CsPbX${}_{3}$ NCs. Mondal et al. found that the hot-exciton cooling process is affected by the pump wavelength, and its lifetime increases with the pump-photon energy (140–700 fs) [14]. However, there are few reports for hot-exciton cooling at high excitation intensity and the AR at different excitation wavelengths to our best knowledge [15,16,17].

In this paper, the AR and the hot-exciton cooling lifetimes of CsPbX${}_{3}$ NCs are investigated by using transient absorption (TA) spectroscopy with different pump fluences and excitation wavelength. The lifetime of hot-excitons increases gradually with the increase of pump fluence and/or with the decrease of excitation wavelength. AR lifetime increases with the decrease of pump fluence. Furthermore, it increases with the increase of the excitation wavelengths.

## 2. Experimental Section

### 2.1. Sample Preparation

CsPbBr${}_{3}$ NCs were synthesized by modifying the hot-injection method [18]. First, 0.069 g of PbBr${}_{2}$ was added to a 3-neck round-bottom flask containing 5 mL of 1-octadecene (ODE). The mixture was degassed at 120 ${}^{\circ }$C for 1 h. Then, 0.5 mL of oleic acid and 0.5 mL of oleylamine were added to the mixture under N${}_{2}$ atmosphere. After 30 min, the temperature of the mixture was raised to 180 ${}^{\circ }$C. The solution of Cs-oleate (0.1 M, 0.4 mL) in ODE was preheated to 100 ${}^{\circ }$C and then added rapidly to the mixture. When the color of the mixture turned green, the reaction was stopped by dipping the reaction flask into an ice bath. At room temperature, 6 mL of n-butanol was added to form CsPbBr${}_{3}$ precipitates, followed by the centrifugation at 7000 rounds per minute (rpm). Finally, wet pellets of the CsPbBr${}_{3}$ NCs were re-dispersed in the n-hexane.

### 2.2. Size Characterization

The morphology of the CsPbBr${}_{3}$ NCs was characterized by using a transmission electron microscopy (TEM, JEOL JEM-2010, Tokyo, Japan) operated at an accelerating voltage of 200 kV.

### 2.3. Spectroscopic Measurements

The steady-state (SS) absorption spectrum was measured by using an ultraviolet–visible–near-infrared (UV–Vis–NIR) spectrophotometer (Cary-5000, Agilent, Palo Alto, CA, USA) at room temperature. The SS-PL spectrum was conducted with an optical fiber spectrometer (USB-4000, Ocean Optics, Dunedin, FL, USA) under the excitation of 400 nm. For the temperature-dependent SS-PL spectrum measurements, a thermostat (LNC-W, Lanhai Instrument LH, Beijing, China) with liquid nitrogen was employed. The data of the PL intensity were collected while the temperature was increasing.

The ultrafast TA spectra were performed using the femtosecond (fs) TA spectroscopy system, which is composed of a regenerative-amplified Ti: sapphire laser system (Coherent, Santa Clara, CA, USA, 800 nm, 35 fs, 7 mJ/pulse, and 1 kHz repetition rate) as the laser source and Helios pump–probe system (Ultrafast Systems LLC, Sarasota, FL, USA) as the spectrometer. The output pulse of the laser source was divided into two parts by the beam splitter. One part entered the optical parametric amplifiers (TOPAS, 800 fs), which generated laser pulses at 325 nm, 365 nm, and 440 nm as pump beams. The probe pulses from 320 nm to 650 nm were generated by focusing another part into the continuously rotating CaF${}_{2}$ crystal. The pump and probe beam overlapped on the sample. After transmitting through the sample, the probe beam was focused into a fiber-coupled spectrometer with CMOS sensors. Time delays (0–8 ns) between the pump and probe pulses were controlled by a motorized optical delay line. The pump beam was chopped by a mechanical chopper rotating at 500 Hz. The instrument response function (IRF) of this system was measured to be 100 fs. The sample was continuously stirred throughout the spectral measurement to suppress the photo-charging effect [19]. All the TA experiments were carried out at room temperature, and the collected data were analyzed by the Surface Xplore software (Surface Xplore 4.2.1, 2019).

The nanosecond time-resolved PL spectra were obtained by detecting the PL attenuation information of the samples at various wavelengths through the grating monochromator (Omni-l300, Zolix, Beijing, China) and oscilloscope (GDS-3354, GWINSTEK, Xinbei, China) with 400 nm excitation wavelength.

## 3. Results and Discussion

**Figure 1:**
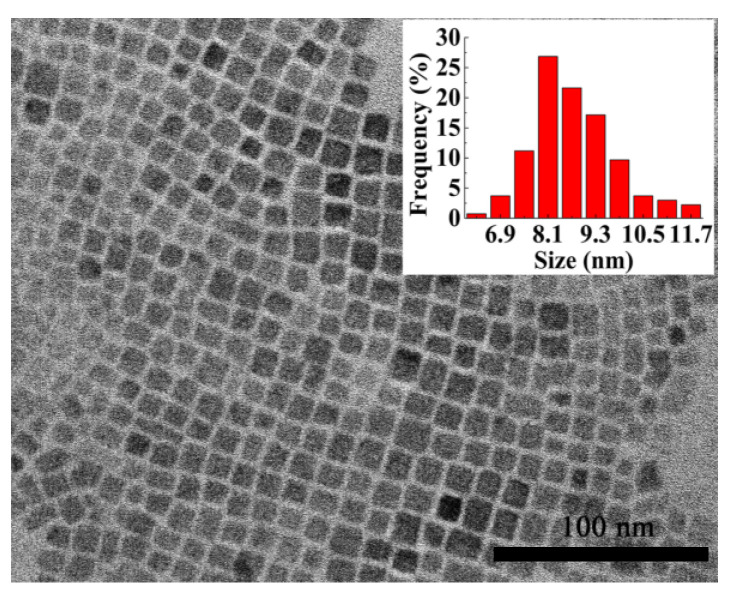
TEM of CsPbBr${}_{3}$ nanocrystals (NCs). The inset shows the size distribution of CsPbBr${}_{3}$ NCs.

The TEM image of CsPbBr${}_{3}$ is shown in Figure 1. The inset displays the size distribution of CsPbBr${}_{3}$ NCs. The average size of CsPbBr${}_{3}$ NCs is ∼8.7 ± 0.5 nm.

**Figure 2:**
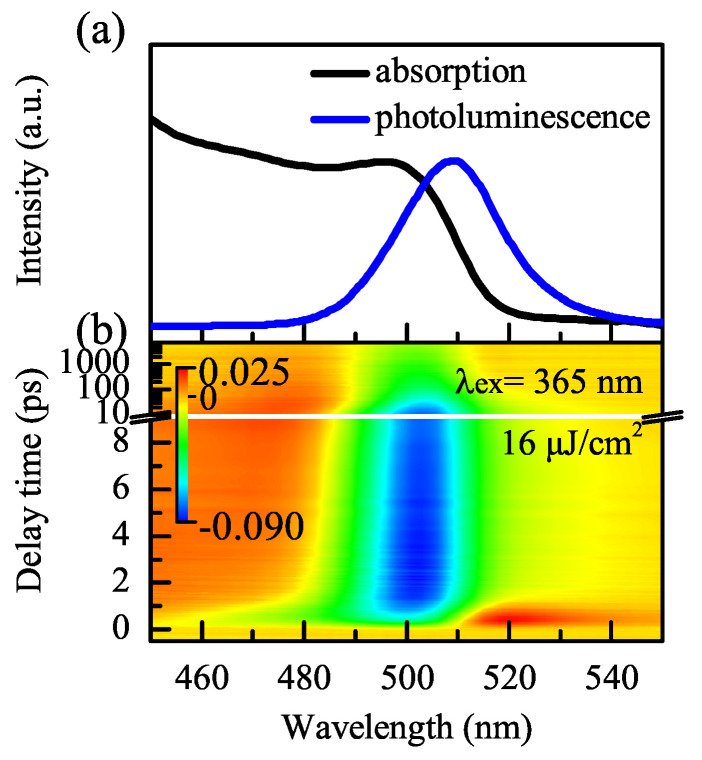
(**a**) SS absorption (black solid line) and PL spectra (blue dashed line) of CsPbBr${}_{3}$ NCs. (**b**) TA contour with an excitation energy of 16 μJ/cm${}^{2}$.

The SS absorption and PL spectra of CsPbBr${}_{3}$ NCs are shown in Figure 2a. As the quantum confinement results in discrete levels, the optically allowed transitions between these levels produce discrete absorption bands in the SS UV–Vis absorption spectrum with the lowest energy exciton band centered at ~495 nm [20]. There is a strong and narrow PL peak at 509 nm, and the Stokes shift is about 44 meV. This Stokes shift is caused by the coupling of the electron transitions in the luminescent centers and vibrations of the CsPbBr${}_{3}$ NCs [21]. Note that the peak position of the PL spectrum is the same whether the initial absorption is pumped at high or low energy. Figure 2b shows the contour of the TA spectrum of CsPbBr${}_{3}$ NCs. There are three features: positive weak broadband in the 450 to 480 nm range (labeled PA1), ground state bleaching (GSB) with a peak at 505 nm, and the second positive absorption band in the 520 to 560 nm region (labeled PA2).

**Figure 3:**
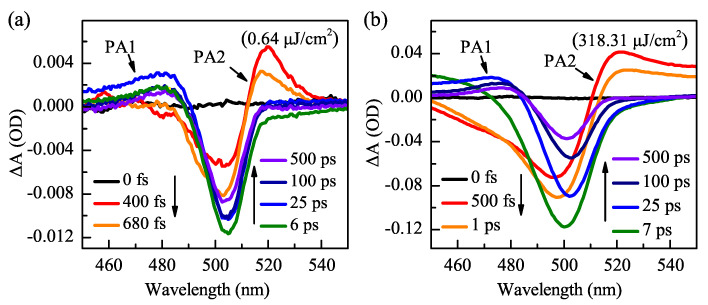
(**a**) TA spectra of CsPbBr${}_{3}$ NCs at the indicated delay time under the lowest pump fluence at 365 nm. (**b**) TA spectra of CsPbBr${}_{3}$ NCs at higher pump fluence at 365 nm.

To measure the carrier dynamics, the ultrafast TA spectral experiments were performed with a pump pulse at 365 nm. The absorbance variation of the detection beam was recorded in the wavelength range of 380 to 650 nm. Figure 3a,b shows TA spectra of the CsPbBr${}_{3}$ NCs with pump fluence of 0.6 μJ/cm${}^{2}$ and 318 μJ/cm${}^{2}$, respectively. The delay time ranges from 0 to 500 ps, and the arrows indicate the evolution of the delay time. The PA1, GSB, and PA2 appear in the TA spectra after the pulse excitation, and these characteristic peaks recovered gradually over time. The PA1 signal is attributed to transitions of newly state-filling excited-state carriers to higher levels [22]. The GSB signal at ∼505 nm is induced by the state-filling of the low energy state of band edges [20,23], corresponding to the first exciton absorption peak in Figure 2a. The GSB amplitude, as well as the bandwidth at low pump fluence of 0.6 μJ/cm${}^{2}$, is smaller than that with a high pump fluence of 318 μJ/cm${}^{2}$. Furthermore, the recovery of the GSB signal in 500 ps is ∼30% at 0.6 μJ/cm${}^{2}$ but 60% at 318 μJ/cm${}^{2}$. This fast GSB recovery process indicates that there is an exciton–exciton extinction process (e.g., non-radiative AR). The PA2 signal at ∼520 nm can be assigned to a transient Stark effect caused by the Coulomb interactions between the hot-excitons and the band-edge excitons [11,24,25,26]. Moreover, the decay of PA2 is accompanied by an increase of GSB from 500 fs to 7 ps. It should be noted that the GSB reaches the maximum around 6 ps, however, the delay time is 500 fs for the PA2 signal.

**Figure 4:**
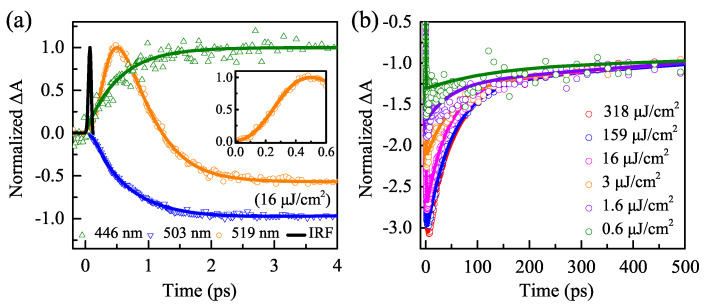
(**a**) TA dynamics detected at the peaks of PA1 (446 nm), PA2 (519 nm), and GSB (503 nm) early scale. Inset: zoomed-in view of the early time for PA2 band. The black-line is IRF. (**b**) Pump fluence dependence of the normalized TA dynamics at the GSB peaks on the long time delay.

Figure 4a shows the TA dynamics with excitation intensity of 16 μJ/cm${}^{2}$ at 446 nm, 503 nm, and 519 nm in the early time, respectively. The inset displays the formation time (∼450 fs) for the PA2 signal. The PA2 signal at 519 nm decays with the buildup of the GSB signal at 503 nm. This phenomenon is caused by the formation of band-edge excitons during the relaxation of hot-excitons [8]. The attenuation time 580 ± 50 fs is obtained by fitting the PA2 signal of 519 nm, corresponding to the hot-exciton intraband cooling-time. The buildup time constant of the GSB signal is 450 ± 20 fs. The PA2 decay time and GSB buildup time are similar, validating the cooling dynamics of hot-excitons, i.e., intraband relaxation [11]. The origin of the GSB signal is attributed to the state-filling effect [20,23]. However, the origin of PA2 is different from GSB because of the difference in the dynamic curves. It is believed that the origin of PA2 is polarons formed by pump-excitons [17]. The PA1 signal at 446 nm is attributed to the absorption arising from the lowest exciton state, as its formation time (520 ± 90 fs) is close to that of GSB [14].

The influence of pump fluence on the bleaching recovery kinetics is conducted for CsPbBr${}_{3}$ NCs. Figure 4b displays the normalized kinetic curves of the GSB peak at 500 ps with different pump fluences. The TA trace with a low-intensity of 0.6 μJ/cm${}^{2}$ is almost flat, meaning that there is only a single-exciton recombination process. The amplitude of the initial TA spectra increases from 1.3 to 3.1 optical density (OD) when the excitation intensity rises from 0.6 to 318 μJ/cm${}^{2}$. Moreover, the amplitude of the initial TA spectra is directly proportional to the number of band-edge excitons generated in the pumping process. The amplitude of the initial TA spectra with the excitation intensity of 318 μJ/cm${}^{2}$ is close to that of 159 μJ/cm${}^{2}$, indicating that the band-edge states are saturated.

The information about the degeneracy of the band-edge states can be extracted. The initial TA amplitude is ~3.1 for the highest pump fluence of 318 μJ/cm${}^{2}$ in Figure 4b. It is ~3.1 times higher than that at 500 ps after the excitation. This implies that the degeneracy for the lowest band-edge states is bigger than two. Makarov et al. found that the band-edges states were twofold degenerate because there are only the lifetimes of biexcitons in the pump fluence-dependent PL and TA spectra for cesium lead halide perovskite QDs [11]. There are biexcitons and multiexcitons with the fluence higher than the threshold value of 1.6 μJ/cm${}^{2}$ generated in CsPbBr${}_{3}$ NCs.

**Figure 5:**
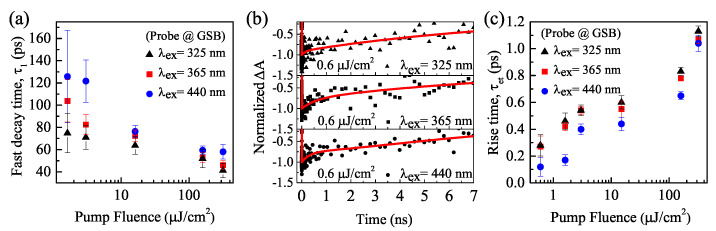
(**a**) Decay time constant of fast decay component the GSB dynamic as a function of pump fluence with 325 nm, 365 nm, and 440 nm pump wavelengths. (**b**) Single-excitons recombination lifetimes with 325 nm, 365 nm, and 440 nm at 0.6 μJ/cm${}^{2}$, and (**c**) rise-time constant of the GSB dynamic as a function of pump fluence with 325 nm, 365 nm, and 440 nm.

The GSB kinetic curves with different excitation intensities at 325 nm, 365 nm, and 440 nm pump wavelengths are fitted using the exponential Formula (1) [27,28],

(1) $$\begin{array}{cc} \Delta A(t) & =a_{1}exp\left(-t/\tau_{1}\right)+a_{2}exp\left(-t/\tau_{2}\right)-c_{1}exp\left(-t/\tau_{et}\right) \end{array}$$

where $a_{1}$, $a_{2}$, and $c_{1}$ are the amplitude; $\tau_{1}$ and $\tau_{2}$ are the decay time constants; and $\tau_{et}$ is the rise-time constant. Decay includes fast and slow components. Figure 5a shows the variation of the fast decay component $\tau_{1}$ with the pump fluences at 325 nm, 365 nm, and 440 nm excitation. As the pump fluence increases from 1.6 to 318 μJ/cm${}^{2}$, the fast decay $\tau_{1}$ decreases from 75 ± 18 ps, 104 ± 19 ps, and 126 ± 41 ps to 41 ± 6 ps, 46 ± 4 ps, and 58 ± 7 ps for 325 nm, 365 nm, and 440 nm, respectively. (The error drops by more than a factor of 2 indicating that the signal-to-noise ratio of the collected data is increased when the pump fluence increases.) It indicates that the fast lifetime $\tau_{1}$ reduces when the pump fluence increases. The fast decay component $\tau_{1}$ is attributed to the non-radiative AR of multiexcitons generated by the absorption of multiple photons in a single NC. Furthermore, the number of photons absorbed by per NC should increase with the increase in excitation intensity, which will amplify the number of excitons, speeding up the AR rate [9,16]. The AR rate can be represented by $dn/dt=-Cn^{3}$, where n is the carrier density and C is the effective Auger constant, and the carrier density-dependent instantaneous AR time ($\tau_{n}$) is described by $\tau_{n}={\left(Cn^{2}\right)}^{-1}$ in bulk semiconductors [12]. For the case of NCs, $\tau_{n}$ is related to the N-exciton Auger lifetime ($\tau_{NX}$) by $\tau_{n}=\tau_{NX}{<N>|}_{<N>=N}$, where *n* is defined as $n=<N>/V_{NC}$ (here <N> is the ratio of the average number of electron–hole pairs per NC, $V_{NC}$ is the NC volume) [29]. The effective Auger coefficient can be expressed as $C=V_{NC}^{2}/\left(8\tau_{2X}\right)$, where $\tau_{2X}$ is the biexciton lifetime [11]. Thus, the Auger constant of CsPbBr${}_{3}$ NCs is obtained between 9.4 × 10${}^{-28}$ to 1.3 × 10${}^{-27}$${\mathrm{cm}}^{6}s^{-1}$ based on the measured lifetime with the pump fluence of 1.6 μJ/cm${}^{2}$. Moreover, the AR lifetime with 440 nm excitation wavelength is much longer than that with 325 nm. It should be noted that there are no fast decay component $\tau_{1}$ at the low pump fluence of 0.6 μJ/cm${}^{2}$, and there is only a single exciton recombination process. The time constant of the slow decay component $\tau_{2}$ caused by the single-excitons recombination is about a few nanoseconds [13,30]. As shown in Figure 5b, the single-excitons recombination lifetimes fitted from the GSB data are ∼7.2 ± 1.2 ns, 6.3 ± 1.1 ns, and 6.8 ± 1.1 ns with pump wavelengths of 325 nm, 365 nm and 440 nm, respectively.

Figure 5c demonstrates the relation between rise-time constant $\tau_{et}$ and pump fluences with excitation wavelengths of 325 nm, 365 nm, and 440 nm for CsPbBr${}_{3}$ NCs, respectively. $\tau_{et}$ is corresponding to the hot-exciton intraband cooling-time. As the pump fluence increases from 0.6 to 318 μJ/cm${}^{2}$, the rise time $\tau_{et}$ increases from 0.28 ± 0.08 ps, 0.27 ± 0.08 ps, and 0.12 ± 0.07 ps to 1.13 ± 0.04 ps, 1.07 ± 0.03 ps, and 1.04 ± 0.06 ps for 325 nm, 365 nm, and 440 nm, respectively. The extension for the hot-exciton cooling time indicates that there is a hot phonon bottleneck [31]. With rising excitation intensity, the number of absorbed optical phonons per NC increases, leading to an increase in the occupancy of the lowest energy levels at the band edge. The hot phonon bottleneck effect slows down the hot-excitons’ relaxation, resulting in the long-lived hot carriers’ population [15,31]. On the other hand, at the same pump fluence, the hot-exciton cooling life $\tau_{et}$ shows an increasing trend with decreasing excitation wavelength. For example, at 0.6 μJ/cm${}^{2}$, $\tau_{et}$ is 0.12 ± 0.07 ps, 0.27 ± 0.08 ps, and 0.28 ± 0.08 ps for the pump wavelengths of 440 nm (2.82 eV), 365 nm (3.40 eV) and 325 nm (3.82 eV), respectively; at 1.6 μJ/cm${}^{2}$, $\tau_{et}$ is 0.17 ± 0.04 ps, 0.42 ± 0.03 ps, and 0.46 ± 0.06 ps for 440 nm, 365 nm, and 325 nm, respectively; at 16 μJ/cm${}^{2}$, $\tau_{et}$ is 0.44 ± 0.05 ps, 0.55 ± 0.06 ps, and 0.60 ± 0.05 ps for 440 nm, 365 nm, and 325 nm, respectively; and at 160 μJ/cm${}^{2}$, $\tau_{et}$ is 0.65 ± 0.03 ps, 0.78 ± 0.02 ps, and 0.83 ± 0.03 ps for 440 nm, 365 nm, and 325 nm, respectively. It means that the time of the hot-carriers cooling to the band edge is increased by increasing the excitation energy. Because the lowest excitation energy (2.82 eV) is close to the bandgap with little excess excitation energy, the GSB signal appears near the time resolution of the IRF (100 fs). However, for the higher excitation energy (3.40 eV or 3.82 eV) well above the bandgap, it generates a larger rising component $\tau_{et}$.

**Figure 6:**
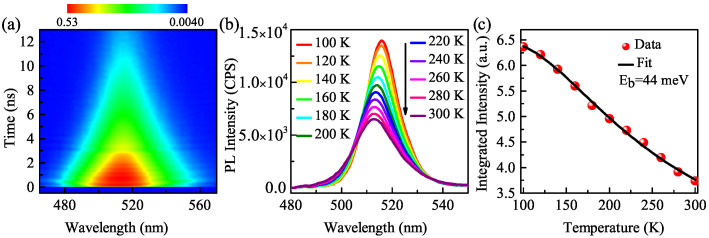
(**a**) Nanosecond time-resolved PL contour. (**b**) Temperature-dependent SS-PL spectra of CsPbBr${}_{3}$ NCs. (**c**) Integrated PL intensity as a function of temperature. The black solid line represents the fit based on the Formula (2).

The fast dynamical processes of CsPbBr${}_{3}$ NCs have been analyzed using TA spectroscopy. To shed more light on the exciton nature of CsPbBr${}_{3}$ NCs, the nanosecond time-resolved PL and variable temperature PL are performed with an excitation wavelength of 400 nm. Figure 6a demonstrates the time-resolved PL contour for the CsPbBr${}_{3}$ NCs. The PL lifetime is 6.9 ns, which matches well with the single-exciton recombination lifetimes (7.2 ± 1.2 ns, 6.3 ± 1.1 ns, and 6.8 ± 1.1 ns) mentioned above. The exciton binding energy, as a key physical parameter of semiconductors, is especially crucial for excitation and recombination during the PL process of inorganic perovskite QDs at room temperature [32]. Figure 6b shows the SS-PL spectra for the CsPbBr${}_{3}$ NCs. The PL peak shifts monotonously from 516 nm to 512 nm as the temperature increases from 100 K to 300 K. The blue-shift of this emission peak is caused by the electron–phonon coupling [33]. It indicates that the band-gap energy increases with the increase of the temperature. On the other hand, the intensity of PL peaks reduced greatly with the increased temperature. This phenomenon is caused by thermally activated non-radiative recombination channels at high temperatures [34]. The dependence of the PL intensity with temperature is shown in Figure 6c fitted with Formula (2) [35],

(2) $$\begin{array}{c} I\left(T\right)=\frac{I_{0}}{1+Aexp\left(-E_{b}/K_{B}T\right)} \end{array}$$

where $I_{0}$ is the integrated PL intensity at 100 K. A is the radiation attenuation constant, $E_{b}$ is the exciton binding energy, and $K_{B}$ is the Boltzmann constant. The fitting yields an $E_{b}$ value of 44 meV, close to the value reported by Li et al. [33]. This value is larger than the thermal disturbance energy at room temperature (≈26 meV), which indicates that CsPbBr${}_{3}$ NCs can generate excitons at room temperature and recombine with a high rate [32].

## 4. Conclusions

The dynamics of hot-exciton cooling and AR for CsPbBr${}_{3}$ NCs is investigated by using the TA spectroscopy. Processes of hot-exciton cooling and AR show a significant dependence on excitation fluence and pump wavelength. With high excitation intensity, the AR dominates the decay kinetics. AR lifetime increases with decreasing pump fluence, and the recombination rate (~126 ps) at 440 nm excitation wavelength is significantly suppressed at low pump power. The hot-exciton cooling time is 0.12–1.13 ps, and the cooling rate increases with the decrease of the excitation intensity and pump-photon energy of the pump light, respectively. The long lifetimes of hot-excitons are achieved by changing the pump light conditions. At low pump fluence, single exciton recombination is the main recombination in CsPbBr${}_{3}$ NCs, and its lifetime is about 7 ns. The exciton binding energy of CsPbBr${}_{3}$ NCs is 44 meV. The dynamics results for multiexcitons interactions and relaxation in CsPbBr${}_{3}$ NCs provide a favorable insight into the applications including solar cells and light-emitting devices based on CsPbBr${}_{3}$ NCs.

## Data Availability

The data presented in this study are available on request from the corresponding author.
